# Relapsed/refractory diffuse large B cell lymphoma with cardiac involvement: A case report and literature review

**DOI:** 10.3389/fonc.2023.1091074

**Published:** 2023-01-30

**Authors:** Yuanyuan Yang, Zixuan Li, Yuntao Li, Yue Zhao, Mingxia Shi

**Affiliations:** ^1^ Department of Hematology, the First Affiliated Hospital of Kunming Medical University, Kunming, China; ^2^ Hematology Research Center of Yunnan Province, Kunming, China; ^3^ Department of Cardiology, the First Affiliated Hospital of Kunming Medical University, Kunming, China

**Keywords:** cardiac hematological malignancy, secondary cardiac lymphoma, B-cell lymphoma, CAR T-cell immunotherapy, case report

## Abstract

**Background:**

Hematological malignancies of the heart (CHMs) are extremely rare, and include leukemia, lymphoma infiltration, and multiple myeloma with extramedullary manifestations. Cardiac lymphoma can be divided into primary cardiac lymphoma (PCL) and secondary cardiac lymphoma (SCL). Compared to PCL, SCL is relatively more common. Histologically, the most frequent SCL is diffuse large B-cell lymphoma (DLBCL). The prognosis of lymphoma in patients with cardiac involvement is extremely poor. CAR T-cell immunotherapy has been recently become a highly effective treatment for relapsed or refractory diffuse large B-cell lymphoma. To date, there are no guidelines that provide a clear consensus on the management of patients with secondary heart or pericardial involvement. We report a case of relapsed/refractory DLBCL that secondarily affected the heart.

**Case presentation:**

A male patient was diagnosed with double-expressor DLBCL based on biopsies of mediastinal and peripancreatic masses and fluorescence *in situ* hybridization. The patient received first-line chemotherapy and anti-CD19 CAR T cell immunotherapy, but developed heart metastases after 12 months. Considering his physical condition and economic situation of the patient, two cycles of multiline chemotherapies were administered, followed by CAR-NK cell immunotherapy and allogeneic hematopoietic stem cell transplantation (allo-HSCT) at another hospital. After achieving a six-month survival, the patient died of severe pneumonia.

**Conclusion:**

The response of our patient emphasizes the importance of early diagnosis and timely treatment to improve the prognosis of SCL and serves as an important reference for SCL treatment strategies.

## Introduction

Secondary cardiac lymphoma (SCL) is a relatively uncommon cancer that affects the heart and/or pericardium, with mortality rates of approximately 8.5% to 25% (1). It is most common in men, especially in immunosuppressed patients, with a median age of 60 years (2, 3). Among cardiac lymphomas, diffuse large B-cell lymphoma (DLBCL) is the most prevalent histology (80%); other histological subtypes are less common (1, 3, 4). The clinical signs and symptoms of SCL tend to be nonspecific, including the superior vena cava (SVC), dyspnea, constitutional complaints, chest pain and B-symptoms (i.e., weight loss, fatigue, night sweats) and so on, resulting in frequently missed diagnoses (1, 2, 4). As secondary cardiac lymphoma is a rare disease, treatment recommendations are largely derived from retrospective studies and case reports (2, 4). Therapies for cardiac lymphoma include mainly chemotherapy, often combined with radiotherapy, surgery, autologous stem cell transplantation, and T cell therapy with the chimeric antigen receptor (CAR) (1, 5, 6). Chemotherapy with cyclophosphamide, vincristine, doxorubicin, and prednisolone (CHOP) is widely used as first-line treatment, but its overall survival rate (OS) is only 18 months, while patients treated with BACOP (bleomycin + doxorubicin + cyclophosphamide + vincristine + prednisone) have a survival of 49 months. Patients receiving surgical resection have a survival of more than 18 months and those with radiotherapy achieve a survival of 15 months (7). The addition of a monoclonal CD20 antibody (rituximab) to the CHOP protocol has shown potential to increase OS rate (1, 8, 9). Despite the fact that these therapies are associated with the greatest improvement in survival, not every patient receives complete multimodal treatment due to individual factors. Therefore, more effective treatment strategies should be developed to improve the outcomes of patients with cardiac involvement. Here, we report a case of relapsed/refractory DLBCL that secondarily affected the heart and was novelly treated with CAR-NK and allo-HSCT.

## Case report

**Figure 1:**
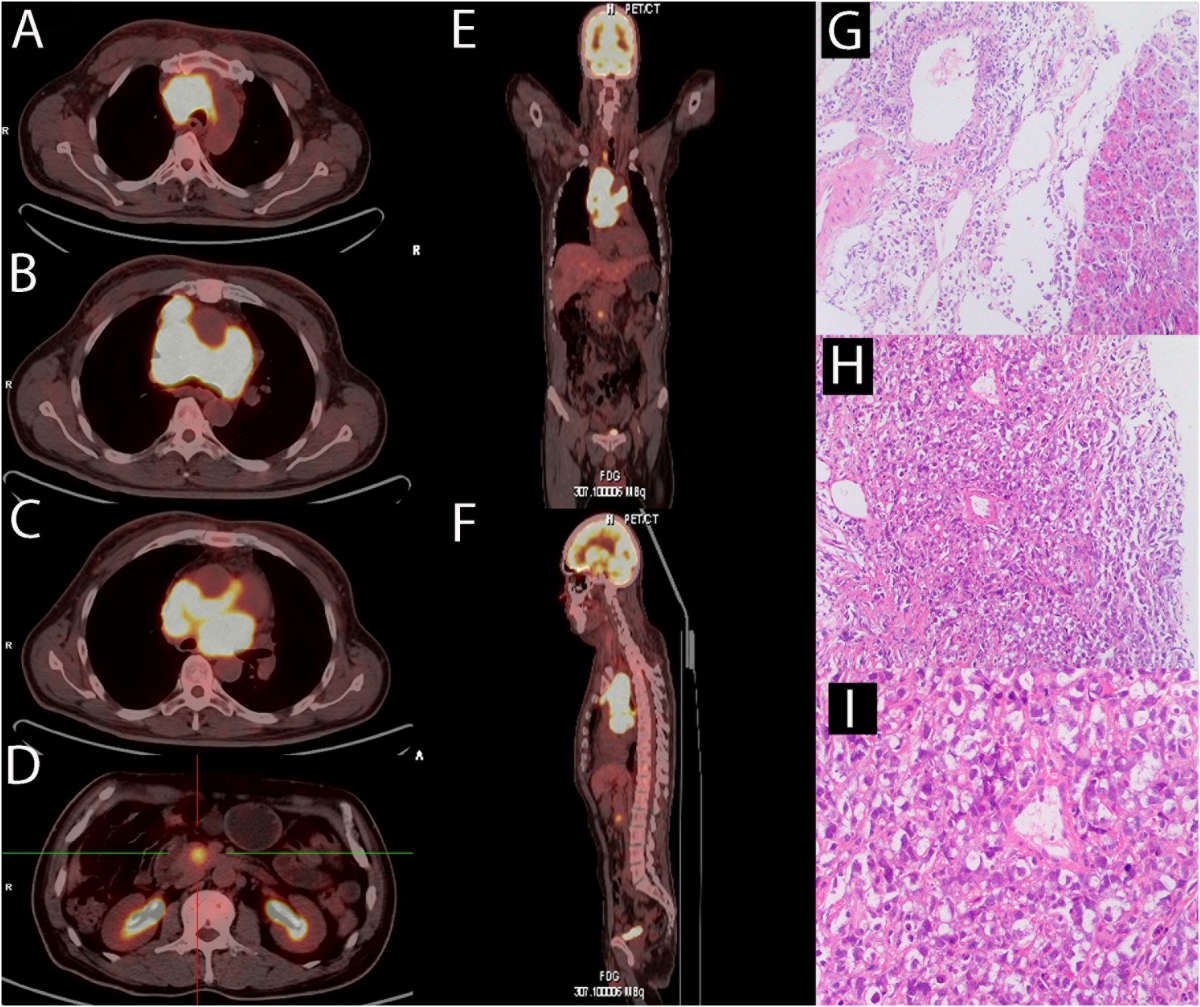
At diagnosis of lymphoma, a PET-CT scan and a biopsy were performed. **(A-C)** PET/CT revealed multiple enlarged lymph nodes in the middle and upper mediastinum and the maximum plane size was approximately 6.3 cm x 4.3 cm. There were masses showing infiltration around the ascending aorta and pulmonary artery. The SUVmax was 13.8. **(D)** PET/CT revealed the uncinate process of pancreas infiltration, with a diameter of about 2 cm and the SUVmax was 5.7. **(E, F)** Overall, there is no significant abnormal radiation uptake outside of the pancreas and mediastinal lymph nodes. **(G)**The hematoxylin-eosin staining revealed medium to large cells with irregular nucleus in the pancreas (original magnification 10 x). **(H, I)** The hematoxylin-eosin staining revealed medium to large cells with irregular nucleus in the mediastinum (H:original magnification 10 x; G:original magnification 20 x).

A 59-year-old man came to our hospital on 10 November 2020, complaining of persistent pitting edema of the face and neck that had been occurring for one month, along with night sweats and backaches, with no weight loss, low fever, dyspnea, cough, pitting edema of the lower extremities, and other symptoms. Laboratory test results are as follows: hemoglobin 114 g/L, platelet count 454×10^9^/L, LDH 519 IU/L, serum β_2_ microglobulin 2.59 mg/L. Chest computed tomography (CT) revealed multiple space-occupying lesions in the middle and upper mediastinum. Positron emission tomography CT (PET-CT) revealed abnormally increased fluorodeoxyglucose (FDG) uptake in the middle and upper mediastinum lymph nodes with a maximum lymph node size of 6.3 × 4.3 cm and the maximum standard uptake value (SUV _max_) was 13.8, the boundary between cardiovascular vessels and masses was not clear (**Figures 1A-C, E, F**). The pancreatic uncinate showed a round focus with high FDG uptake of approximately 2.0 cm in diameter, the SUV_max_ was 5.7 (**Figures 1D-F**). On 18 November 2020, a CT-guided percutaneous pancreas and mediastinum biopsy was performed. Hematoxylin-eosin staining of pancreatic mases (**Figure 1G**) and mediastinum (**Figures 1H, I**) showed medium to large abnormal lymphocytes with irregular nucleus. Immunohistochemical staining revealed that tumor cells were positive for CD19 (>95%), CD3, CD20, CD30, BCL-6, PAX-5, c-Myc, Ki-67 (90%+), and was negative for Syn, CgA, CK, CA199, CK20, CK7, CD56, and BCL-2. Both bone marrow cytology and biopsy results revealed no aberrant lymphocyte infiltration. In summary, the patient was diagnosed with DLBCL at stage IVB. The IPI score was 3 and the ECOG performance status was 1. The immunohistochemistry results revealed a non-germinal center B-cell-like lymphoma (non-GCB) subtype. The patient had no family history of hematological malignancies.

**Figure 2:**
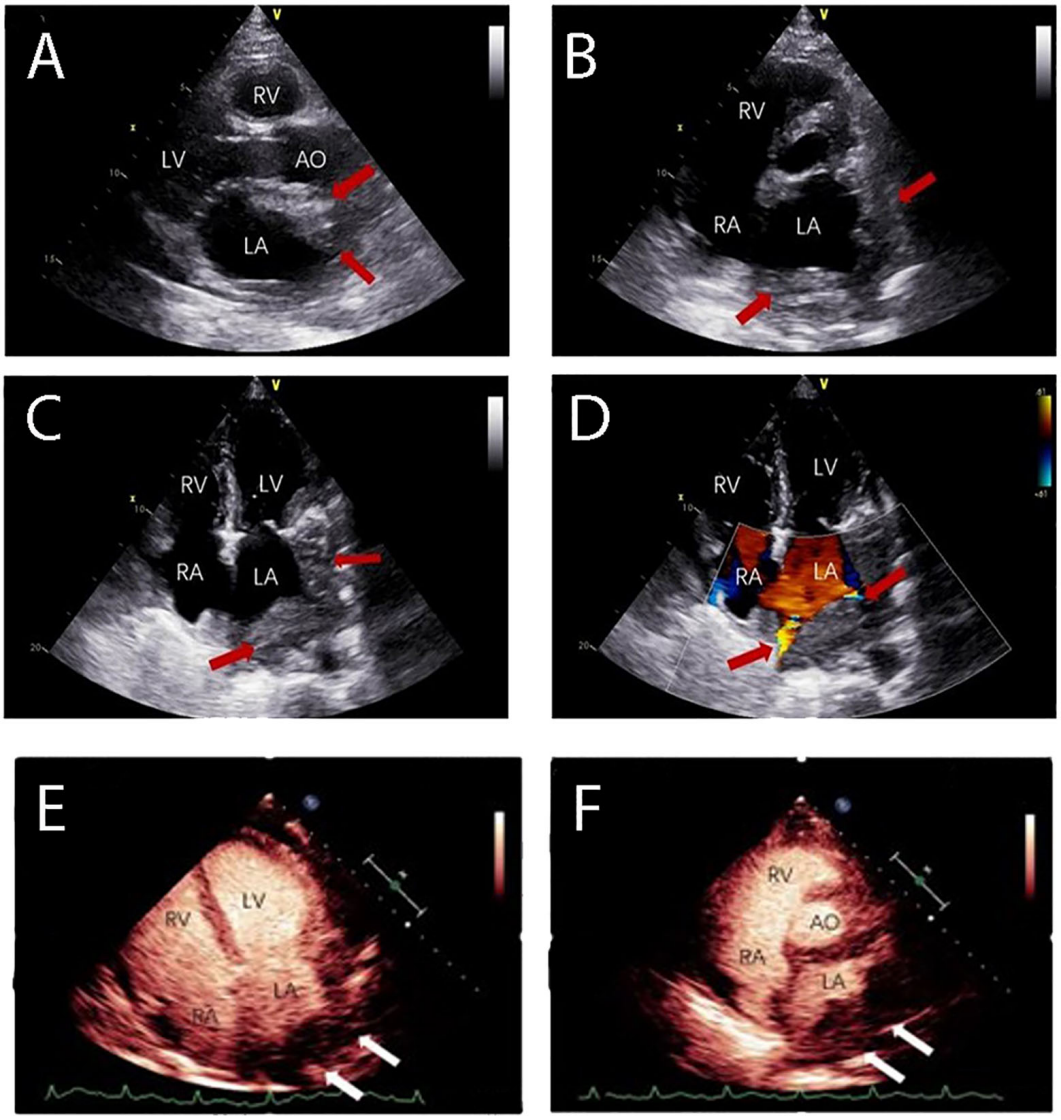
The patient experienced dyspnea and had a refractory bilateral pleural effusion after receiving multi-line chemotherapy. Transthoracic echocardiography showed the heart infiltrated by lymphoma. **(A)** Long axis section of left ventricle showing a slight hypoechoic mass in the anterior wall of the left atrium. **(B)** Lateral wall of left atrium and left atrial appendage were infiltrated. **(C, D)** Four-chamber view showed pulmonary vein infiltration. Cardiac ventricular opacification (CVO): **(E, F)** An irregular mass was present at the entrance of pulmonary veins in the left atrium with rapid irregular perfusion and complete enhancement. (LV: Left Ventricle; RA: Right Atrium; RV: Right Ventricle; LA: Left Atrium).

**Figure 3:**
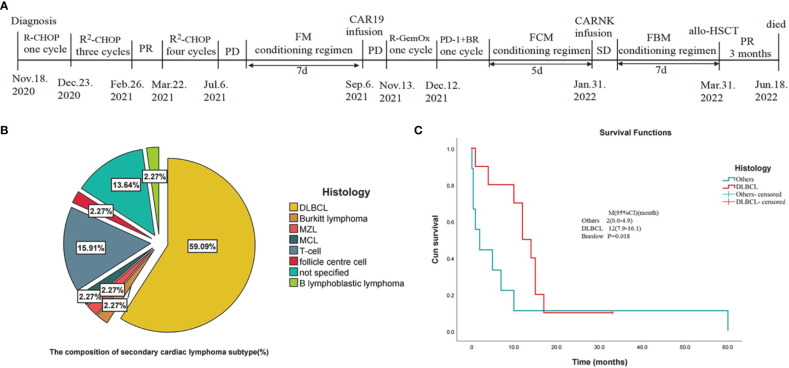
Statistical analysis of the data in the literature review (10–48) and the timeline of disease status and corresponding treatment regimens of our patient. **(A)** Timeline of disease status and corresponding treatment regimens. **(B)** Pie chart of tissue type composition of secondary cardiac lymphoma. (DLBCL, diffuse large B-cell lymphoma; MZL, marginal zone lymphoma; MCL, mantle cell lymphoma; T-cell: T cell lymphoma). **(C)** Kaplan-Meier survival curves for secondary cardiac lymphomas comparing DLBCL with other cancers included in the statistics. (CI, confidence interval).

The patient was started on induction chemotherapy with one course of R-CHOP (rituximab 375 mg/m^2^ on day 0, doxorubicin 30 mg/m^2^ and cyclophosphamide 750 mg/m^2^ on day 1, vincristine 2 mg on day 1, and prednisolone 60 mg/m^2^ on days 1–5) and three courses of R^2^-CHOP (rituximab 375 mg/m^2^ on day 0, doxorubicin 30 mg/m^2^, cyclophosphamide 750 mg/m^2^, and vincristine 2 mg on day 1, prednisolone 60 mg/m^2^ on days 1–5, and lenalidomide 25 mg on days 1–21), he achieved partial remission by PET-CT. The patient then received two separate apheresis procedures, including granulocyte colony-stimulating factor (G-CSF)-stimulated autologous hematopoietic stem cell (HSC) collection (CD34^+^ 4.35×10^6^/kg). Followed by three more courses of R^2^-CHOP. Unfortunately, although the patient had no obvious symptoms, PET-CT showed progressive disease. To prevent the progression of the disease, he was transferred to another institution for CAR T cell therapy. The patient was given a standard dose of FC (Fludarabine 25mg/m^2^ on days -7 to -5, cyclophosphamide 250 mg/m^2^ on days -7 to -5) as conditioning regimen one week before CAR-T cell therapy. On 6 September 2021 (day 0), CD19-targeted CAR-T cells (CAR19) (2.0x10^7^ cells/kg) were infused into the patient. One month after CAR-T cell therapy, a second contrast-enhanced chest CT during follow-up showed that lymph nodes in the middle and upper mediastinum were larger than before. Shortness of breath, palpitations, hypoxemia, and fever ensued. Combined with the history of the disease and PET-CT, he was considered to have a progressive disease. On PET-CT, the largest lymph node was approximately 8.3 cm and the SUV_max_ was 13, the masses fused in the pulmonary gaps and their boundaries were unclear, the Deauville score was 5. He had pneumonia and refractory bilateral pleural effusion; bilateral drainage was performed as a salvage procedure. Cytology, biochemistry, and flow cytometric analysis of the pleural fluid did not show any infiltration of abnormal lymphocytes. As a result of antibiotic therapy, pleural chest drainage, and supportive therapies, the patient’s symptoms improved. R-GemOx (gemcitabine 1000 mg/m^2^ and oxaliplatin100 mg/m^2^ on day 1, and rituximab 375mg/m^2^ on day 0) was initiated on 13 November 2021. Three weeks later, the patient’s symptoms began to worsen, and the pleural fluid was sent for a circulating tumor DNA (ctDNA) test, which revealed somatic hypermutation (MYC, SOCS1, IGLL5, BTG1, DTX1, PIM1) and high-frequency mutation (TET2, IL4R, ACTG1, B2M, SGK1, HIST1H1E), indicating malignant pleural effusion. Transthoracic echocardiography left ventricular contrast echocardiography (LVO) and myocardial contrast echocardiography (MCE) detected heart metastases (**Figures 2A-F**). All clinical evidence indicated that the patient’s dyspnea and refractory bilateral pleural effusion were due to cardiac metastases; the possibility of thrombi was excluded. The patient was then treated with one cycle of PD-1+BR (sintilimab 200 mg on day 0, rituximab 375 mg/m^2^ on day 1, bendamustine 90 mg/m^2^ on days 2–3). After that, he was eager to undergo a CAR-T clinical trial for the second time. Unfortunately, at this time his T cell counts were very low. He was then treated with CAR-NK immunotherapy. He was administered the FCM regimen (fludarabine 30 mg/m^2^ on days -5, -4, -3, cyclophosphamide 300 mg/m^2^ on day -5, melphalan100 mg/m^2^ on day -4) regimen five days before CAR-NK. On 31 January 2021 (day 0), CAR-NK cells (5.6x10^6^ cells/kg) were infused into the patient. On+7d he experienced grade 2 cytokine release syndrome (CRS) and was treated with tocilizumab 8 mg/kg, after which his symptoms improved. One month after CAR-NK immunotherapy, patient white blood cells and lymphocytes did not graft and the copy numbers of the CAR-NK transgene decreased. He experienced a transient reduction in pleural effusion and relief of dyspnea, chest CT showed that the cardiac mass was smaller than before which indicated stable disease. We then informed the patient and his family about his condition and treatment plan, and both signed consent forms for allogeneic hematopoietic stem cell transplantation. He received FBM (fludarabine 30mg/m^2^ on days -7 to -2d, busulfan 3.2 mg/kg on days -7, -6, and melphalan100 mg/m^2^ on day -2). Graft-versus-host disease (GVHD) prophylaxis consisted of anti-thymocyte globulin 2 mg/kg on day -1, cyclophosphamide 30 mg/kg on days +3 and +4, dexamethasone 40 mg/kg on days +3 and +4, ruxolitinib 5 mg twice a day from on days -1 to +50, 2.5 mg twice a day on days +51 to +100, and 2.5 mg once a day on days +101 to +180, myfortic 720 mg twice a day on days +5 to +34, ciclosporin 15 mg/kg/d (adjusted according to blood concentration), methotrexate 5 mg/m^2^ on days +3 and +6d. On 31 March 2022 (day 0), the patient underwent haploid allogeneic hematopoietic stem cell transplantation (CD34^+^ 6.35×10^6^/kg), the donor was his son. Leukocytes and platelet engraftment occurred on day +16. After allo-HSCT, the patient’s condition continued to remain stable. 100% donor chimerism was achieved. On 30 April 2022, chest CT showed no new lesions and his pleural effusion decreased significantly. The timeline of clinical treatment and the state of the disease are shown in **Figure 3A**. However, during this period, he experienced severe fungal pneumonia. Despite multiple antifungal, antibacterial, and antiviral treatments, the patient eventually died from septic shock on 18 June 2022.

## Discussion

Cardiac hematological malignancies (CHM), which comprise leukemic, lymphoma infiltration, and extramedullary manifestations of multiple myeloma, are rarer than any other malignant cardiac tumors (3). Primary and secondary cardiac lymphomas are of two varieties (10). Primary cardiac lymphomas, which comprise approximately 0.5% of all lymphomas and approximately 1.3% of all primary cardiac malignancies, are lymphomas that primarily affect the heart and/or pericardium (49). Secondary cardiac lymphomas, which are 20–40 times more prevalent than initial cardiac lymphomas, account for approximately 5%–20% of disseminated diseases (1, 50). Approximately all cardiac lymphomas have B-cell origins, and DLBCL are the most common type (80%) (1). In a retrospective investigation, DLBCL (58%), T-cell lymphoma (16%), Burkitt lymphoma (9%), and small lymphocytic lymphoma (6%) were the most prevalent histologic subtypes of NHL with biopsy-proven cardiac involvement (4). Here, we reviewed 44 patients with secondary cardiac lymphoma published on PubMed and CNKI between 1976 and 2022 (**Table 1**). Patients with SCL had an average age of 55 years. SCL is more common in men than in women (64.3% vs 35.7%). DLBCL was the most prevalent NHL subtype in patients with morphologic evidence of cardiac involvement (**Figure 3B**), accounting for 59.09% of cases, followed by T-cell lymphoma (16%) and Burkitt’s lymphoma (2.27%). There is a chance of direct, lymphatic, or hematogenous heart metastases (51).

**Table 1:** Clinical characteristics of patients with cardiac lymphoma reported to date (10–48).

Characteristics	
Age, n (%)	
16-60	21 (47.7)
>60	23 (52.3)
Male sex, n (%)	27(64.3)
Lymphoma type, n (%)	
B cell	31(70.4)
T cell Not specified	8(18.2) 5 (11.4)
Previous HIV/AIDS, n (%)	
Yes	17(38.6)
No	7 (15.9)
Unknown	20(45.5)
Clinical symptoms, n (%)	
Dyspnea	23(63.9)
Fatigue	5 (13.9)
Abdominal pain	4(11.1)
Fever	4(11.1)
Cough	3(8.3)
Chest tightness	3(8.3)
Palpitation	3(8.3)
Dysphagia	2(5.6)
Weight loss	2(5.6)
Night sweat	2(5.6)
Others	7(15.6)
Mass localization in the heart, n (%)	
Left atrium	6(14.3)
Left ventricle	7(16.7)
Right atrium	17(40.5)
Right ventricle	7(16.7)
Tricuspid valve	3(7.14)
Epicardium	1(2.3)
Pericardium	2(4.8)
Atrial septum	3(7.14)
Endomyocardium	1(2.3)
Septal myocardium	1(2.3)
Right myocardial wall	1(2.3)

Since cardiac lymphoma often does not present symptoms or abnormal behavior, its diagnosis is often delayed (51, 52). Depending on the location, size, growth rate, degree of invasion, and friability of the tumor, patients with cardiac involvement present with a variety of symptoms (10, 53). Based on our review of the literature, 44 cases of secondary cardiac lymphoma were located (**Table 1**). More specifically, in decreasing order of frequency, the right atrium, right ventricle, left atrium, and left ventricle were affected more frequently. The patient may have symptoms of right heart failure or superior vena cava syndrome (SVC) if a tumor is found in the right atrium or right ventricle (10). However, shortness of breath is often seen if a large mass occurs in the left atrial or left ventricular region (10). The most common clinical symptom described is dyspnea (64%), followed by constitutional problems (26%) and chest pain (24%) (11, 54). In our review (**Table 1**), the results are the same. In this case, our patient presented dyspnea, left facial swelling, and tachycardia. This was associated with lymphoma that affected the superior vena cava (SVC) and the pulmonary vein, causing limited venous inflow, which is called SVC syndrome (5–8%) (1). As a result, he was diagnosed with secondary cardiac lymphoma.

When regard to detecting cardiac involvement, ECG and chest radiographs are generally insensitive or nonspecific (51, 55, 56). A transthoracic echocardiogram is the first non-invasive method used to examine the heart and pericardium; however, the small acoustic window of this technology remains a major drawback (53). A study that included PCL patients found that transesophageal echocardiography (TEE) had higher sensitivity than transthoracic echocardiogram (TTE) for the diagnosis of lymphomatous involvement (97% vs 75.9%) (51, 57). With a high degree of spatial and temporal resolution, cardiac masses can be diagnosed by computed tomography (CT) based on their distribution, shape, and size (1). Early detection and treatment of cardiac abnormalities, as well as tracking of chemotherapy response, are now possible thanks to PET/CT (1, 51). The optimum imaging technique to determine whether the malignancy has affected the heart is CMRI (1, 51). Cardiovascular tumors show hypointense in T1-weighted sequences, while appearing hyperintense in T2-weighted sequences (11, 51). If there is a possibility of cardiac involvement, magnetic resonance imaging should be chosen despite the fact that these various imaging modalities should be considered complementary rather than competitive (53, 54).

The prognosis for cardiac lymphoma tends to be extremely poor (1). These neoplasms are often not recognized until postmortem due to their nonspecific symptoms (1). Gordon et al. found that patients with primary cardiac B-cell NHL had better outcomes than those with secondary cardiac involvement (2 months versus 6 months) (4). We found that DLBCL secondary cardiac lymphomas had a better median survival time (MST) compared to non-DLBCL secondary cardiac lymphomas (12 vs 2 months) (**Figure 3C**). It is important to note that patients who are immunocompromised, have extracardiac disease, left ventricular involvement, and do not have an arrhythmia are the most important adverse prognostic factors (58). Treatment guidelines are not available (58); thus, the treatment of cardiac lymphoma is varied. The medical literature presents a variety of treatment options, including chemotherapy, radiotherapy, surgery, and even autologous stem cell transplantation (1). However, it should be noted that chemotherapy is the most effective treatment and, in many cases, is used only for palliative purposes (2). The main chemotherapeutic regimen for cardiac lymphoma is CHOP (1). Adding the monoclonal CD20 antibody rituximab and other monoclonal therapies has been shown to increase overall survival rates (1). Cardiac lymphoma is also sensitive to radiotherapy (59). Radiation is, however, restricted to cardiac masses that progress despite chemotherapy due to cardiovascular side effects (2). Yang et al. suggested that the overall response rate to secondary cardiac lymphoma was 63.2% and the median survival time was 18 months. Petrich et al. reported that the overall response rate of primary cardiac lymphoma to chemotherapy was 79% and complete remission was 59% (57). Similar results have been reported for secondary lymphoma (57). The management of patients with R/R DLBCL has improved significantly in the last year (60). Various novel antibodies, ADCs, specific small-molecule inhibitors, as well as CAR-T cells, have been approved for the treatment of affected patients (61, 62). The outlook for cardiac lymphoma remains poor (1).

## Conclusion

Lymphoma metastases to the heart are rare and are associated with a poor prognosis. We report a case of secondary cardiac lymphoma and analyze published case reports. Unfortunately, our patient was unable to undergo an endomyocardial biopsy due to his poor physical condition. Despite the rapid changes in currently available treatment options, the prognosis for cardiac lymphoma remains poor. Various treatments appeared to have improved our patient, but he unfortunately died of severe pneumonia in the end. Therefore, early diagnosis and timely treatment are still significant for improving survival. And our experience has shown that early allo-HSCT has an irreplaceable role in the survival benefit of patients. When an individual has a history of recurrent pleural effusions, dyspnea, and lymphoma, cardiac infiltration should be considered. To improve patient survival, additional treatment options should be explored.

## Patient perspective

Despite the death outcome, the encouragement and optimism of the patient’s family (particularly his son) inspired him to confront the disease. Throughout the treatment procedure, he showed a determined will to survive. He was willing to attempt a variety of treatments that may have been more effective but also riskier. With regard to the disease and treatment, he and his family expressed gratitude to our hospital and doctors.

## Data availability statement

The original contributions presented in the study are included in the article/supplementary material. Further inquiries can be directed to the corresponding author.

## Ethics statement

Written informed consent was obtained from the individual(s) for the publication of any potentially identifiable images or data included in this article.

## Author contributions

YY wrote the manuscript and analyzed the data. MS, YY, and ZL revised the manuscript and processed images. YL, MS, YY, and ZL diagnosed and treated the patient. YZ operated cardiac ultrasound and elaborated the picture of cardiac ultrasound. MS funded the research. All authors contributed to the article and approved the submitted version.
